# Gut microbiota: emerging biomarkers and potential therapeutics for premature ovarian failure

**DOI:** 10.3389/fmicb.2025.1606001

**Published:** 2025-07-15

**Authors:** Zongyu Liu, Min Wang, Yuanyuan Lei, Kaiqi Xu, Limei Fan

**Affiliations:** ^1^Department of Surgery, The Second Hospital of Jilin University, Changchun, China; ^2^Department of Obstetrics and Gynecology, The Second Hospital of Jilin University, Changchun, China; ^3^Medical Imaging Technology Program, Jilin Medical University, Jilin, China

**Keywords:** gut microbiota, premature ovarian failure, premature ovarian insufficiency, infertility, biomarkers, therapeutics

## Abstract

Premature ovarian failure is a prevalent gynecological endocrine disorder with an increasing incidence rate each year, impacting women’s physical and mental health. The causes of POF are poorly understood, but genetic, immune, iatrogenic, environmental, and psychological factors are key contributors. Clinically, POF manifests as oligomenorrhea, amenorrhea, elevated follicle-stimulating hormone (FSH) levels, and decreased estrogen levels, leading to infertility in women. POF not only impacts reproductive function but also elevates the risk of cardiovascular diseases, osteoporosis, depression, anxiety, cognitive decline, and neurological disorders, thereby adversely affecting women’s mental health and quality of life over the long term. The gut microbiota (GM) comprises a vast and complex microbial community within the human gastrointestinal tract. GM dysregulation is closely associated with numerous human diseases, including autoimmune diseases, allergic disorders, cardiovascular diseases, cancers, and metabolic disorders. Studies have shown that GMs play a pivotal role in female reproductive health, participating in the pathogenesis of reproductive endocrine disorders through direct or indirect involvement in sex hormone regulation, stimulation of inflammatory cytokine production, modulation of immune function, metabolic homeostasis, and regulation of neurotransmitter synthesis. Recently, advancements in human microbiology have highlighted the significant interest in the connection between POF and the gut microbiome. Researching the molecular mechanisms by which GMs and their metabolites regulate the occurrence of POF opens up a new direction for studying the pathogenesis of POF. This research aims to identify an efficient, non-invasive, and accurate diagnostic method for clinical diagnosis and treatment of POF, providing novel theoretical insights and precise intervention strategies for the clinical prevention and treatment of POF.

## 1 Introduction

**FIGURE 1:**
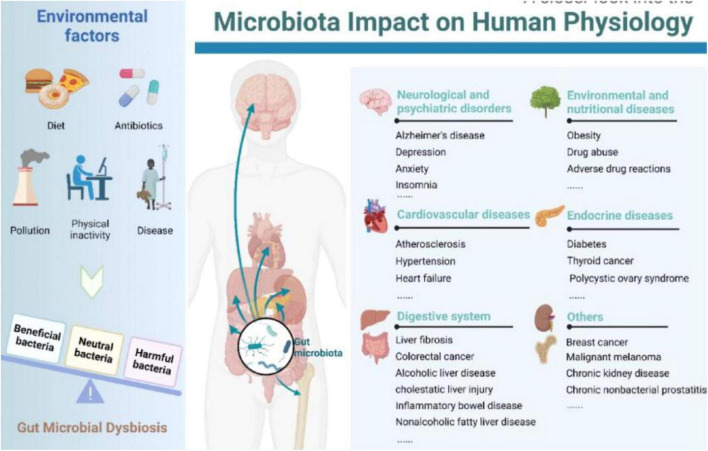
Relationship between gut microbiota and humans (Zuo et al., 2023). Left: Multiple environmental factors, encompassing diet, antibiotics, pollution, physical inactivity, and disease, may engender perturbations in the gut microbiota. Right: Gut microbiota aberrations have been linked to a myriad of morbidities, spanning neuropsychiatric disorders, cardiovascular afflictions, gastrointestinal ailments, diseases arising from environmental and nutritional factors, endocrinopathies, and more.

The ovary, one of women’s most vital organs, maintains female fertility and endocrine functions. The ovary is susceptible to various factors, including age, genetics, immunity, medical interventions, environment, dietary structure, and psychosocial factors, which can impair ovarian reserve and function through direct or indirect pathways (Caserta et al., 2013; de Souza et al., 2015; Schuh-Huerta et al., 2012; Várbíró et al., 2022). Ovarian reserve indicates ovarian follicles’ remaining quantity and quality, representing a woman’s reproductive potential (Hussein, 2005). Infertility is a highly prevalent global condition, and the prevention and treatment of female infertility face severe challenges, with no effective means currently available to slow this trend. Despite the rapid advancement of human *in vitro* fertilization-embryo transfer technology, clinicians face challenges, including low oocyte retrieval, poor embryo quality, high cycle cancelation, and low clinical pregnancy rates. Diminished ovarian function is currently a research hotspot in gynecological reproductive endocrinology (Gulan et al., 2019). POF refers to ovarian dysfunction occurring before age 40 in women. The main clinical manifestations of POF include decreased menstrual flow, prolonged menstrual cycles or even amenorrhea, elevated FSH levels, and decreased estrogen levels (Sullivan et al., 2016). In the 21st century, with the launch of the “Human Microbiome Project,” trillions of microorganisms have received increasing attention, particularly the most complex GM (Cullin et al., 2021; Puschhof et al., 2021). Advances in genome sequencing technology and bioinformatics have elucidated the impact of GM on health and the interaction between microorganisms and their hosts (Sender et al., 2016; Zuo et al., 2023; Figure 1). The GM parasites are on the surface of the gastrointestinal tract, with most not being pathogenic and coexisting with intestinal epithelial cells. The gut microbiota plays crucial roles in host nutrient metabolism, preserving intestinal mucosal barrier integrity, regulating the immune system, and defending against pathogens (Qin et al., 2010). Research findings suggest that GM dysregulation contributes to the onset and progression of multiple reproductive endocrine disorders. The gut microbiota affects seminal fluid microbiota in adult male mice on a high-fat diet, influencing semen quality (Ding et al., 2020). In patients with polycystic ovary syndrome, the composition of the GM is disrupted, and the diversity and abundance of the GM improve significantly after drug treatment (Torres et al., 2019). Consequently, the GM can affect POF occurrence and progression through various pathways and factors. Researching the relationship between the GM and POF holds great potential and value for application, providing new diagnostic and therapeutic approaches for POF.

## 2 Overview of POF

Premature ovarian failure refers to ovarian dysfunction occurring in women before age 40, accompanied by high gonadotropin (Gn)-induced amenorrhea due to follicular depletion. The main clinical manifestations include oligomenorrhea or amenorrhea, elevated FSH levels, and fluctuating decreases in estrogen levels (Nash and Davies, 2024). Diminished ovarian reserve (DOR) primarily refers to a reduction in the number or quality of oocytes within the ovaries, accompanied by decreased anti-Müllerian hormone levels, decreased antral follicles, and elevated FSH levels (Cedars, 2022). Clinically, this may present as menstrual disorders, reduced flow progressing to amenorrhea, recurrent miscarriages, and infertility, impacting women’s reproductive health and quality of life. Furthermore, it can progress to premature ovarian insufficiency (POI) (Torrealday and Pal, 2015). POI is the early stage of ovarian failure, occurring in women before age 40, characterized by decreased ovarian function with serum hormone changes such as elevated FSH levels and low estrogen levels. The primary features include menstrual disorders, including amenorrhea or frequent menstruation (Shi et al., 2023). Patients often experience Gn-excessive amenorrhea due to estrogen deficiency, accompanied by an increased incidence of osteoporosis, cardiovascular disease, depression, cognitive dysfunction, and other conditions (Eastell, 2003; Roeters van Lennep et al., 2016; Tao et al., 2016).

The postponement of the childbearing age has become a global issue. With the increase in work pressure on women, environmental changes, and various other factors, the incidence of POF has shown an upward trend year by year. It is affecting younger women (American College of Obstetricians and Gynecologists Committee on Gynecologic Practice and Practice Committee, 2014; Yang Q. et al., 2021). The pathogenesis of POF remains unclear, and its etiology is complex and diverse, involving genetic factors, age, iatrogenic factors, autoimmune factors, endocrine disorders, infectious factors, environmental factors, psychosocial factors, and lifestyle habits (D’Avila et al., 2015; Pelosi et al., 2015; Szeliga et al., 2021; Titus et al., 2015). Autoimmune factors account for up to 10%–30% of the causes of POF and are related to the regulation of multiple cytokines (Jagarlamudi et al., 2010). The majority of POF patients experience perimenopausal symptoms such as facial flushing, hot flashes, and hyperhidrosis. POF adversely affects women’s reproductive health and imposes psychological burdens while also elevating the risk of osteoporosis, cardiovascular diseases, and other conditions, thereby impairing patients’ quality of life and overall health (Allshouse et al., 2015; Francucci et al., 2008). Currently, the primary treatment for POF is Hormone Replacement Therapy (HRT). Although HRT can alleviate clinical symptoms such as hot flashes and hyperhidrosis caused by hormone deficiency, it does not fundamentally address the damage to ovarian structure and function. Relapses are common after discontinuation, and long-term hormone use increases the risk of developing diseases such as endometrial cancer, breast cancer, and thrombosis (Lambrinoudaki and Paschou, 2021). Therefore, exploring effective and safe treatment options for POF is an urgent clinical issue (Schover, 2008).

## 3 Overview of GM

The microbiome is one of the hottest research areas in biomedicine, related to various life processes in the human body, such as metabolism, immunity, reproduction, neurology, and inflammation. The composition of microorganisms in biological samples is demonstrated through sequencing, classification, and statistical analysis of the microbiome using 16S ribosomal RNA and metagenomic sequencing techniques, revealing its high diversity (Gopalakrishnan et al., 2018; Lee et al., 2024; Yang et al., 2019). GM is a crucial component of human microecology, and due to its vast number and powerful functions, it is known as the “second genome” of humans. The intestinal microecology encompasses the gut microbiome and its environment, where bacteria, fungi, and viruses in the human gastrointestinal tract coevolve and coexist with the host, forming the intestinal microbiota (de Vos et al., 2022). There are over 1,000 known bacterial species in the human gastrointestinal tract, and the number of GM is ten times that of human cells, with genes encoded by GM being 150 times that of the human genome. Through interactions with the environment, GM can influence the body’s physiological metabolism and pathological processes (Makki et al., 2018). GM is distributed segmentally along the digestive tract, with the lowest bacterial count in the stomach and most colonization occurring in the colon and ileum. As an essential component that coexists with the body for a long time, a delicate balance has been formed between GM and the host (Coyte and Rakoff-Nahoum, 2019). GM plays a significant role in the pathophysiological processes of human growth, development, metabolism, immunity, etc., including promoting the maturation of the host’s immune system, inhibiting pathogen overgrowth, influencing host cell proliferation and angiogenesis, regulating intestinal endocrine function, neurotransmitter signaling, energy sources, and vitamin and neurotransmitter synthesis (Cani, 2018; Xu et al., 2024). GM influences the intestinal environment and regulates distant tissues and organs, functioning as a mature endocrine organ.

### 3.1 Composition of GM

**FIGURE 2:**
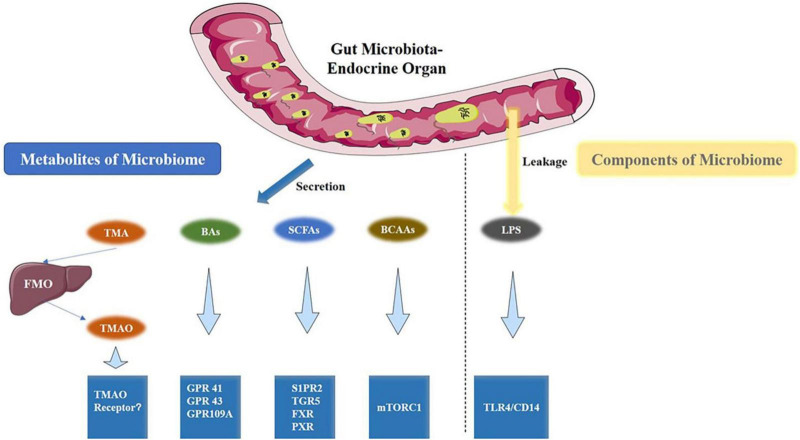
Gut microbiota metabolites and the associated metabolic signaling pathway (Yang Q. et al., 2021). TMAO, BAs, SCFAs, and BCAAs are the metabolites that are produced by the gut microbiota. Conversely, LPS is a component of the cell wall of gut bacteria. All are capable of activating specific signaling pathways. BAs, bile acids; BCAAs, branched-chain amino acids.

The human GM primarily comprises four phyla: Firmicutes, Bacteroidetes, Proteobacteria, and Actinobacteria, with Bacteroidetes and Firmicutes being the most dominant (Parker et al., 2020; Sun et al., 2021). Firmicutes have immune-regulating effects, while Firmicutes, Bacteroidetes, and *Ruminococcus* influence the production and metabolism of estrogens. Studies have reported that Verrucomicrobia is a species of significant research value, within which *Akkermansia* belongs, and is considered to play a crucial role in the human intestinal microbiota, exhibiting probiotic properties (Gómez-Gallego et al., 2016). The Lachnospiraceae family plays a vital role in intestinal health by engaging in the digestion and metabolism of dietary fiber, carbohydrates, and sugar transport. Recent studies have highlighted metabolites such as short-chain fatty acids (SCFAs), trimethylamine N-oxide (TMAO), bile acids, polyphenols, and indoles as being closely linked to host health (Vallianou et al., 2019; Yang X. et al., 2021; Figure 2). TMAO, produced by the metabolism of choline in food by intestinal microorganisms, is a key factor in inducing atherosclerosis. This discovery has advanced research on the relationship between intestinal microbial metabolites and extraintestinal organ damage (Koeth et al., 2013). Changes in these differential microbiotas, such as Firmicutes, Bacteroidetes, Verrucomicrobia, Proteobacteria, Lachnospira, and *Ruminococcus*, and their compositional structure may be one of the pathogenic mechanisms of ovarian dysfunction (Lin et al., 2019).

### 3.2 Metabolites of GM–SCFAs

Short-chain fatty acids, mainly acetic, propionic, and butyric acids, constitute over 95% of the total (O’Riordan et al., 2022). As messengers between the GM and the host, SCFAs exert extensive physiological effects on the host (Shen et al., 2019). They preserve intestinal barrier integrity, reduce colon pH, suppress harmful bacterial growth, and are involved in glucose and lipid metabolism (Ji et al., 2019). *Lactobacillus* and *Bifidobacterium*, recognized as beneficial bacteria, exert their beneficial effects by producing SCFAs and are widely used in probiotic preparations. SCFAs are essential for immune regulation and promoting inflammatory responses in pathological processes (LeBlanc et al., 2017). They can directly or indirectly inhibit LPS-activated TLR-4/NF-κB pathways, increase the expression of local cellular pro-inflammatory factors, and downregulate IL-8 and MCP-1, thereby achieving localized pro-inflammatory and overall anti-inflammatory effects (Lin et al., 2015; Segain et al., 2000). SCFAs regulate the distribution and localization of zonula occludens-1 and occludin in the intestinal barrier by influencing the endocannabinoid system, reducing the entry of endotoxin into the bloodstream, and thus controlling inflammation, intestinal barrier function, and intestinal peptide secretion (Muccioli et al., 2010). The endocannabinoid system is associated with female infertility, participating in the physiology and pathophysiology of the endometrium and placenta, influencing the successful implantation of blastocysts and the maintenance of early pregnancy, and holding a pivotal position in human reproduction (Maia et al., 2020). SCFAs affect oxidative damage in reproductive organs through autophagy-related pathways, influencing processes such as the origin of oocytes, the formation of ovarian reserve, follicle recruitment, and the selection of dominant follicles, which are crucial for follicular development (Kumariya et al., 2021). SCFAs also influence the hypothalamic-pituitary-ovarian (HPO) axis. In female obese rats, they can notably reverse precocious puberty, inhibit hypothalamic gonadotropin-releasing hormone (GnRH) secretion, and postpone gonadal axis development via the Kiss1-GPR54-PKC-ERK1/2 pathway (Wang et al., 2022).

### 3.3 The function of GM

Gut microbiota forms a mutualistic symbiotic relationship with the host and participates in various biological processes of the host through multiple mechanisms, including digestion and metabolism of food, development, and immunity (Rudi and Zhao, 2021). Changes in the composition of related microbial species and alterations in internal environmental homeostasis may trigger associated diseases (Wan et al., 2022). The function of the gut microbiota is not limited to the intestine; it can secrete various cytokines, induce inflammation and metabolic changes, and affect the immune system and neuroendocrine system functions (Melamed et al., 2022). The secretion and production of metabolites are the primary ways in which GM participates in the pathophysiological processes of the host. These metabolites can exert local effects in the intestine or enter the blood circulation through the intestinal barrier to affect the functions of multiple extraintestinal organs (Wang et al., 2019). GM can regulate the activity of various enteroendocrine cells and affect the gut-brain axis function. The gut-brain axis can further influence the structure of GM through the immune system (Cheng et al., 2023). GM influences systemic diseases via immune, neuroendocrine, and metabolic pathways (Manor et al., 2020). The human microbiome holds great potential in disease prevention and control: firstly, the composition or functional genes of the human microbiome can be used to predict the occurrence, progression, and outcome of related diseases; secondly, interventions and adjustments can be made to the microbiome to achieve disease prevention and treatment goals (Helmink et al., 2019; Matson et al., 2021; Weersma et al., 2020).

### 3.4 Balance of GM

Gut microbiota generally maintains a dynamic balance, interacting with the host and influencing the occurrence and progression of diseases by regulating the metabolism of substances such as sugars, lipids, and amino acids. Imbalances in gut microbiota may contribute to the onset of associated diseases (Takeuchi et al., 2023). Like other organs, the normal function of GM depends on its stable composition (Hörmannsperger et al., 2012). GM dysbiosis is primarily driven by environmental and host-related factors, such as antibiotic use, changes in dietary structure, immune activation, and stress states (Janczyk et al., 2007; Mountain, 1988). GM dysbiosis occurs when these factors disrupt the gut microbial ecosystem beyond its resistance and recovery capabilities, with effects ranging from transient to permanent and from benign to harmful (Fassarella et al., 2021). GM dysbiosis is mainly classified into three types: (1) Overproliferation of pathogens. In a healthy gut, pathogens exist at relatively low abundances, but in intestinal infection and inflammation states, Enterobacteriaceae bacteria can overproliferate; (2) Reduce or complete loss of commensal bacteria. This situation can be improved by exogenous supplementation of commensal bacteria. For example, in the case of *Clostridium difficile* infections, colonization with *Clostridium scindens* can improve the severity of the disease; (3) Decreased gut microbiota diversity. The impact of GM on reproductive organs is primarily through circulatory transport, where GM products or product-induced endocrine, oxidative stress, and inflammatory factors are transported to the reproductive organs, affecting reproductive function (Dey and Ray Chaudhuri, 2023; Yang et al., 2022).

## 4 The GM is intricately linked to the onset and progression of reproductive system diseases

**FIGURE 3:**
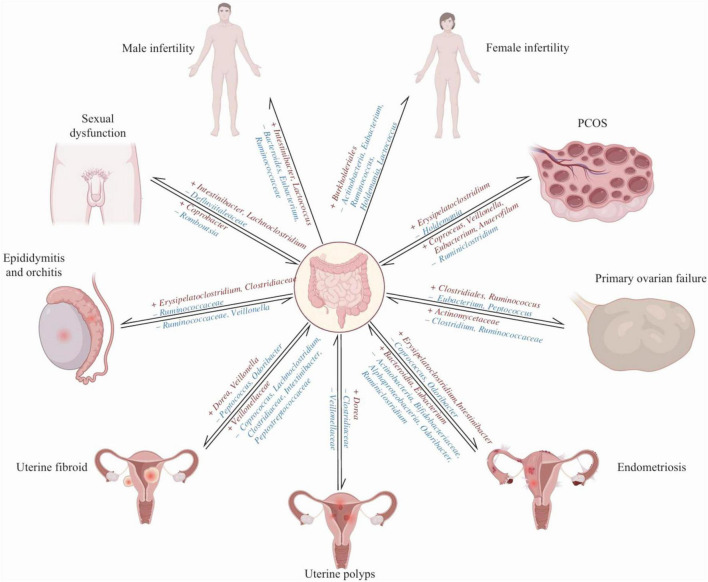
A bidirectional causal relationship between the gut microbiota and infertility, as well as associated diseases (Zhang et al., 2024). The protective bacteria are denoted in blue font (–), whereas the pathogenic bacteria are represented in red font (+).

The GM is closely related to the occurrence and progression of reproductive system diseases (Figure 3; Zhang et al., 2024). The endocrine levels of the ovaries can indirectly reflect ovarian function. The HPO axis controls hormone secretion. The hypothalamus secretes GnRH to stimulate the pituitary, which then releases FSH. This hormone enhances estrogen secretion by acting on the ovaries. Elevated estrogen levels negatively feedback on GnRH and FSH to maintain hormonal balance. Estrogen and FSH are key indicators for evaluating ovarian function. Estrogen, a type of hormone secreted by mature follicles, promotes the proliferation and differentiation of granulosa cells, regulates their apoptosis, and modulates steroid hormone levels. FSH primarily promotes the differentiation and maturation of granulosa cells in follicles and controls follicle development. Elevated FSH levels can lead to premature follicle maturation and early ovulation, resulting in follicular depletion. When estrogen secretion levels are too low, and FSH secretion levels are too high, it can be used to initially assess ovarian reserve function, which is essential for diagnosing POF (Brown et al., 2010). Corresponding vitamin deficiencies can affect sperm production and follicular development (Salam et al., 2015). Pyridoxine can regulate the synthesis and secretion of estrogens and progesterone, affecting the menstrual cycle and ovarian function. It can prevent preeclampsia and preterm birth and increase fertility rates (Wacker et al., 2000). Vitamin B12 is essential for oocyte maturation and quality, with deficiencies impacting ovarian function and oocyte development (Kim et al., 2020). Lipopolysaccharide (LPS), a key bacterial cell wall component in the gut, is crucial for maintaining intestinal barrier integrity, nutrient absorption, gut microbiota balance, and systemic inflammation (Płóciennikowska et al., 2015). One of the most common causes of reproductive disorders is LPS entering the circulation and triggering systemic inflammation and oxidative stress (Agarwal et al., 2017).

### 4.1 The GM influences ovarian function via the HPO axis

Gut microbiota is intrinsically linked to ovarian function. The composition and metabolites of gut microbiota can affect the regulation of the HPO axis, thereby influencing the growth and development of ovarian tissue and oocytes (Hwang et al., 2015). Metabolites like SCFAs and bile acids from GM effectively regulate hypothalamic GnRH neuron function (Liao et al., 2021). GnRH is a key pathway in regulating reproductive function. GnRH secreted by the hypothalamus binds to pituitary receptors, stimulating the release of luteinizing hormone (LH) and FSH, LH and FSH stimulate the gonads to synthesize and secrete steroid hormones like testosterone, estrogen, and progesterone, facilitating the development of target organs. Ovarian aging is closely related to mitochondrial dysfunction, and oxidative stress-induced mitochondrial dysfunction is essential to ovarian aging. Mitochondria are crucial for cellular energy metabolism, the cell cycle, and signaling (May-Panloup et al., 2016). There is a dialogue between GM and mitochondria, and mitochondria can induce granulosa cell apoptosis and autophagy, leading to follicular atresia and premature ovarian failure (Bajpai et al., 2018; Thevaranjan et al., 2017; Yadav et al., 2018).

The gut-brain axis serves as a bridge for bidirectional communication between GM and the central nervous system. GM dysbiosis can alter the release of neuroendocrine hormones and affect neurotransmitter activity, subsequently influencing the central nervous system (Shekarabi et al., 2024). Research indicates that reduced levels of Firmicutes and Bacteroidetes can elevate serum glucagon-like peptide-1 (GLP-1) and its expression, which stimulates GnRH neurons. It modulates neurons and the neurotransmitter gamma-aminobutyric acid (GABA) to regulate GnRH secretion (Outeiriño-Iglesias et al., 2015). GM can produce most neurotransmitters in the brain, such as GABA, dopamine, norepinephrine, acetylcholine, and serotonin (5-HT) 0.5-HT can increase cAMP levels in immature oocytes, promoting oocyte meiotic maturation (Stricker and Smythe, 2001). As a precursor of melatonin, 5-HT can divert tryptophan away from the melatonin pathway through the kynurenine pathway, thereby reducing circulating melatonin levels (Davidson et al., 2022; Fernstrom, 2016). Melatonin may postpone ovarian aging by acting as an antioxidant, preserving telomeres, enhancing SIRT expression and ribosomal function, and decreasing autophagy. It safeguards oocytes from oxidative damage and enhances the expression of genes involved in fatty acid β-oxidation and mitochondrial biogenesis, supplying essential energy for oocyte maturation and embryonic development (Jin et al., 2017; Tamura et al., 2017).

## 5 POF and GM

### 5.1 GM characteristics of POF patients

**FIGURE 4:**
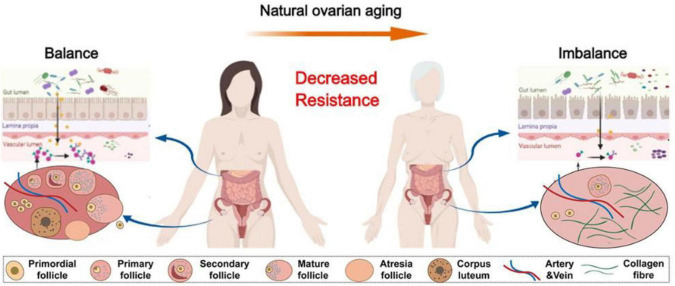
The physiologic succession of gut microbiota across natural ovarian aging (Huang et al., 2024).

Gut microbiota in patients with POF exhibits significant imbalances, and these alterations are associated with the occurrence and progression of the disease (Huang et al., 2024; Figure 4). Cao et al. (2020) in a POF mouse model, a significant increase in gut microbiota α-diversity was observed compared to normal mice. POF mice exhibited lower *Helicobacter*, *Odoribacter*, and *Alistipes* but higher *Clostridium XIVa*, *Barnesiella*, *Bacteroides*, and *Mucispirillum* (Cao et al., 2020). A comparison of gut microbiota revealed that healthy women had higher Firmicutes, *Ruminococcus*, and *Caldibacillus* levels.

In contrast, POF patients exhibited a greater abundance of Bacteroidetes, *Butyricimonas*, *Dorea*, *Lachnobacterium*, and *Sutterella* (Wu et al., 2021). Differences exist in the gut microbiota of patients with POI compared to a standard control group, with differential bacteria such as *Bifidobacterium*, *Bacillus*, and *Clostridium* being able to produce SCFAs in the human intestine (Wang et al., 2023). Not only does the gut microbiota of POF patients change, but their reproductive tract microbiota also undergoes alterations. Wang et al. (2018) study found notable differences in the diversity and richness of vaginal microbiota between POF patients and healthy women. The relative abundance of lactic acid bacteria was notably lower in POF patients than in postmenopausal women (Wang et al., 2018).

### 5.2 The mechanism of GM disorder participating in POF

Current research suggests that the relationship between GM and POF is mainly reflected in autoimmune responses, participation in sex hormone regulation, oxidative stress, inflammatory reactions, and ovulation disorders (Sharif et al., 2019). The GM acts as a messenger in the interaction between the host and the external environment, not only maintaining normal intestinal barrier function, resisting the invasion of pathogenic bacteria, participating in the digestion and absorption of nutrients to provide energy for the host, but also producing important metabolites and promoting the maturation of immune cells (Schluter et al., 2020). The GM can mitigate POF by modulating the expression of immune-related cytokines, including regulatory T cells (Treg), interferon-γ (IFN-γ), and T helper 17 cells (Th17) (Colliou et al., 2018; Motedayyen et al., 2018; Yin et al., 2018b). The GM is also broadly involved in hormone metabolism, bone density, and central nervous system regulation, all of which are closely related to the etiology and clinical symptoms of POF. GM’s development prospects and research space are vast, and an in-depth investigation of the relationship between GM and POF holds significant clinical value.

#### 5.2.1 GM and humoral immunity, cellular immunity

Autoimmune abnormalities are one of the crucial factors contributing to the development of POF. A systemic pro-inflammatory state affects the ovarian environment, and autoimmunity can attack ovarian tissue, impacting follicular growth and development, causing follicular atresia and ovarian atrophy, directly leading to decreased ovarian function. Disruptions in gut microbiota can influence immune cytokines, producing primary ovarian failure POF (Kirshenbaum and Orvieto, 2019). Gomez de Agüero et al. (2016) the study observed transient colonization of the gut microbiota in pregnant rats, identifying it as a key factor in stimulating immune system development in their offspring, thereby suggesting that maternal gut microbiota influences the offspring’s immune system. In the host immune system, GM plays a crucial role. If GM is disrupted, the immune surveillance function of the body decreases, leading to immune escape and disease occurrence. Studies have found an imbalance in the expression of Th1/Th2 cytokines in T lymphocyte subpopulations in POF patients (Lu X. et al., 2019). Some scholars believe that the immune cells CD4 + T/CD8 + T may be related to Th and Treg cells. An increased or decreased CD4 + T/CD8 + T ratio in POI patients reflects a close correlation between POI pathogenesis and T-cell subpopulation imbalance and immune suppression (Kobayashi et al., 2019).

Gut microbiota can affect Treg, IFN-γ, and Th17, and there is a correlation between POF and these immune components (Britton et al., 2019). The potential connections between POF, GM, and immune cytokines include a correlation involving GM, POF, and Treg cells (Song et al., 2018). GM enhances Treg cell expression and differentiation, facilitating their role in anti-inflammatory responses and influencing immune and metabolic homeostasis. Clinically, post-treatment POF patients exhibit Treg numbers and enhanced immune regulation alterations. GM, POF, and IFN-γ are correlated. GM treatment may influence serum IFN-γ levels, which, along with genetic stimulation, can enhance MHC class II antigen expression. This process might trigger autoimmune responses, resulting in follicular atresia and the development of POF (Coulam and Stern, 1991). A correlation exists among GM, POF, and Th17. The study observed that hMSC transplantation in POF mice led to a reduction in Th17/Tc17 and Th17/Treg cell ratios, lower serum IL-17 levels, and decreased granulosa cell apoptosis compared to POF mice treated with a PI3K/Akt inhibitor. This suggests that the PI3K/Akt signaling pathway might play a role in restoring ovarian function by balancing Th17/Tc17 and Th17/Treg cells (Yin et al., 2018a). Therefore, decreased ovarian function is closely related to immune cytokines.

#### 5.2.2 GM participates in hormone metabolism

##### 5.2.2.1 The effect of GM on estrogen

**FIGURE 5:**
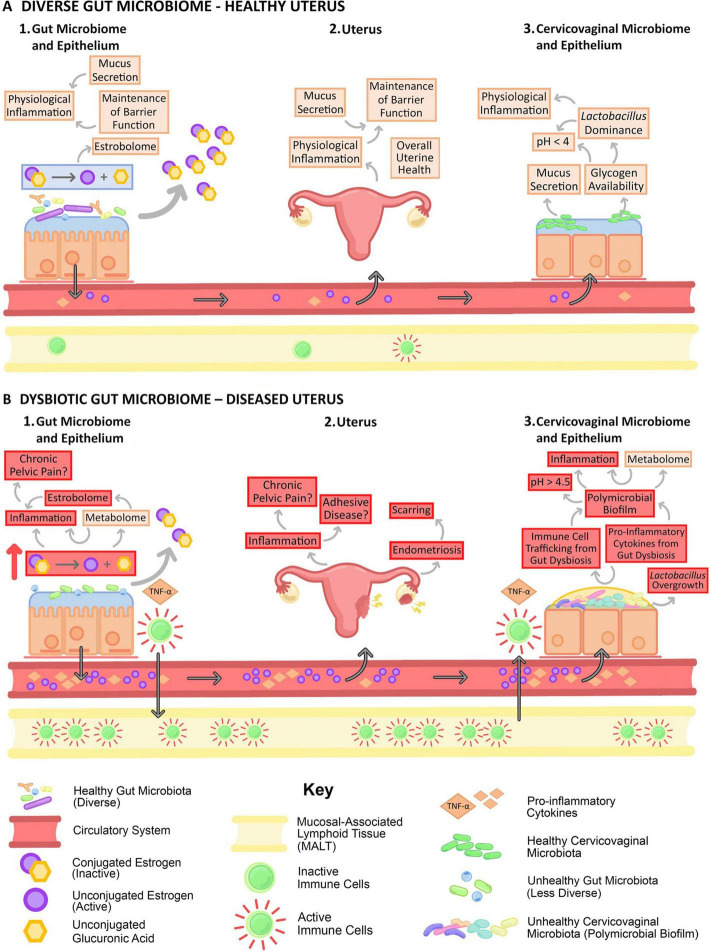
Gut dysbiosis negatively impacts the estrobolome (Salliss et al., 2021). Gut dysbiosis negatively impacts the estrobolome and the female reproductive tract by altering circulating estrogen levels, increasing systemic inflammation, and immune cell trafficking.

Estrogens influence the gut microbiota’s structure and impact estrogen levels, playing a role in estrogen-regulated diseases through the estrogen-GM axis (Salliss et al., 2021; Figure 5). GM influences ovarian function through estrogen levels (Bates, 2018; García-Peñarrubia et al., 2020). Estrogens are crucial for female reproductive development and maintenance. Estrogens are major regulators of the GM, which is influenced by estrogens and positively affects estrogen levels. The GM capable of metabolizing estrogens possesses a gene pool known as the “estrogenome” (Baker et al., 2017). James and other scholars suggest that gut microbiota contributes to the development of estrogen-dependent diseases by introducing the “estrogen-gut microbiota axis,” which influences estrogen levels via β-glucuronidase secretion. β-Glucuronidase deconjugates estrogens, allowing them to bind to estrogen receptors and trigger physiological effects (Noguera-Julian et al., 2016). Altered gut microbiota composition and reduced diversity diminish β-glucuronidase activity, resulting in lower active estrogen levels (Plottel and Blaser, 2011). Reduced circulating estrogens change the activation of estrogen receptors, further contributing to various reproductive endocrine disorders. Estrogens promote follicular development directly and influence ovarian function indirectly by exerting negative feedback on Gn release through the HPO axis. Decreased estrogen levels are one of the essential factors in ovarian function decline. Recent studies indicate alterations in the gut microbiota of POI patients, accompanied by reduced estrogen levels. After adjusting for body mass index and performing Pearson correlation analysis, correlations were found between specific gut microbiota and serum estradiol, FSH, LH, and anti-Müllerian hormone levels (Wu et al., 2021).

Studies have shown that altering GM through antibiotic use can lead to contraceptive failure and unintended pregnancies among women using oral contraceptives (Adlercreutz et al., 1984). Consuming yogurt to improve GM, primarily by increasing GABA and isoflavone levels, is beneficial for health (Fayed, 2015). Isoflavones are the most common “plant estrogens,” structurally and functionally similar to human estrogens. Research results indicate that altering the GM of ovariectomized rats leads to menopause-related symptoms due to decreased estrogen levels (Jeong et al., 2017). Prolonged intake of a symbiotic formulation with *Lactobacillus fermentum* and β-glucan can alleviate menopause symptoms due to estrogen deficiency in rats. The possible mechanisms of action are: ① β-Glucan can comprehensively stimulate the immune system, promote the production of immunoglobulin M antibodies, and enhance humoral immunity. β-Glucan positively influences gastrointestinal microecology by fostering beneficial intestinal bacteria growth and removing harmful substances. It also contributes to lowering cholesterol and low-density lipoprotein levels while enhancing high-density lipoprotein levels (Lewis-Barned et al., 2000).

##### 5.2.2.2 The effect of GM on androgens

Androgen levels are one of the fundamental prerequisites for women’s health (McHenry et al., 2014). Androgen deficiency can cause sexual dysfunction symptoms, including reduced libido and diminished sexual response (Davis and Wahlin-Jacobsen, 2015). Women with POI not only lack estrogens but may also experience reduced ovarian androgen production due to ovarian cortical atrophy (Gleicher et al., 2013). A meta-analysis reveals that women with POI or POF are at risk of reduced levels of total testosterone, dehydroepiandrosterone sulfate, androstenedione (Soman et al., 2019). Androgens can improve the early stages of follicular development and effectively enhance ovarian reserve in women with DOR (Gleicher et al., 2011). Studies have found that low testosterone levels are essential steps involved in the occurrence of endometriosis combined with POI (Ono et al., 2014). In women with DOR, baseline testosterone levels are positively correlated with pregnancy outcomes following *in vitro* fertilization (Lu et al., 2014). There is a close relationship between the GM’s composition and circulating testosterone levels. Prenatal exposure of female rats to testosterone cypionate results in decreased abundances of *Akkermansia*, *Bacteroides*, *Lactobacillus*, and *Clostridium* in adult female offspring, suggesting an interaction between androgens and the gut microbiota (Sherman et al., 2018; Yurkovetskiy et al., 2013). The exact role of gut dysbiosis in the pathogenesis of androgen-dependent metabolic disorders deserves further investigation (Markle et al., 2013; Mayneris-Perxachs et al., 2020).

#### 5.2.3 GM and oxidative stress and inflammatory responses

Gut microbiota dysbiosis may contribute to DOR development by inducing oxidative stress and chronic inflammation. Studies have confirmed that one of the reasons for increased oxidative stress in the body is GM dysbiosis, and oxidative stress has also been proven to be associated with DOR (Zou et al., 2020). In women with DOR, total granulosa cells are induced by oxidative stress to undergo increased apoptosis, leading to poorer ovarian response and reduced oocytes (Fan et al., 2019). Reactive oxygen species accumulation in oocytes leads to mitochondrial dysfunction, endoplasmic reticulum stress, and meiotic maturation disorders, ultimately impairing oocyte maturation and quality (Jungheim et al., 2010; Wu et al., 2010). The imbalance between elevated pro-inflammatory cytokines (TNF-α, IL-6) and reduced anti-inflammatory cytokines (IL-4, IL-10) contributes to POI (Huang et al., 2019). Studies have found that some ovarian tissue pathological biopsies from patients with POF show follicular inflammatory cell infiltration (Sun et al., 2018). Prebiotics help restore gut microbiota balance, decrease oxidative stress, and enhance the expression of anti-inflammatory and antioxidant molecules (Vasquez et al., 2019).

#### 5.2.4 GM dysbiosis and ovulatory dysfunction

Hiroyuki et al. found that the relative abundance of butyrate-producing bacteria decreased in women with chronic anovulation. In contrast, the relative abundance of *Prevotella* increased, suggesting that GM dysbiosis could contribute to ovulatory dysfunction (Sasaki et al., 2019). *Allobaculum* can utilize carbohydrates to produce butyrate, increase SCFAs, enhance glucuronidase activity, reduce estrogen excretion, regulate hypothalamic neurons, and improve ovarian function (Flores et al., 2012). The abundance of *Hungatella* bacteria is positively correlated with taurine levels in the blood, and after the increase of *Hungatella*, taurine levels in the blood return to normal (Li et al., 2020). Taurine may activate the TGR5 protein-mediated AKT signaling pathway, promote ovarian tissue repair, and protect ovarian tissue (Lu M. et al., 2019). Fecal microbiota transplantation from women with ovulatory dysfunction to normal mice resulted in ovarian dysfunction and impaired fertility (Qi et al., 2021). GM dysbiosis can lead to oocyte developmental disorders, disruption of estrus cycles, and abnormal ovulation, and in the future, improving fertility may be achieved by regulating GM.

### 5.3 GM and complications of POF

#### 5.3.1 GM and bone metabolism

The incidence of osteoporosis in women is generally higher than in men (Gregson et al., 2022). The prevalence of osteoporosis in women is primarily attributed to ovarian aging, diminishing follicular reserves, reduced oocyte quality, and lower serum estrogen levels (Touraine, 2020). Estrogen influences bone remodeling by altering cytokines and growth factors within the bone marrow and bone cells (Warden et al., 2010). Recent bone microbiota studies have also linked changes in bone phenotypes to changes in the GM. The hypothesis that a “healthy” microbiota is necessary to prevent sex hormone deficiency-induced bone loss is supported by evidence showing that supplementing the diet of ovariectomized mice with probiotics can reverse the pathogenic process of osteoporosis (Lyte, 2011). Additionally, several studies assessing the bacterial quantity and diversity in the intestines of patients with osteoporosis indicate that adult osteoporosis reduces biodiversity, such as species within the genera *Fusobacterium*, *Faecalibacterium*, and *Bacteroides* (Gustafsson et al., 2006; Yan and Charles, 2017).

#### 5.3.2 GM and mental psychology

Psychological factors are one of the main contributors to POF. As a difficult-to-treat gynecological disease, POF not only causes abnormal menstruation, hot flashes, vaginal dryness, and other discomforting symptoms but may also induce psychological issues such as depression, anxiety, tension, and decreased self-confidence (Haller-Kikkatalo et al., 2015). Research has demonstrated that maintaining gut microbiota balance is essential for regulating brain behavior and cognitive function (Cossu et al., 2021). The interaction between gut microbiota and the central nervous system is crucial in developing mental disorders. The GM participates in basic neurobiological activities such as blood-brain barrier formation, myelination, neurotransmitter transmission, and glial cell maturation and regulates various animal behaviors (Li et al., 2022). Symbiotic bacteria, probiotics, and pathogens in the digestive tract, as initiators of a mild inflammatory state, can stimulate central nervous system pathways and signaling systems, causing dysfunction in the “microbe-gut-brain axis” and thus exacerbating the neuropsychiatric problems of POF patients (Kohn et al., 2021).

### 5.4 Chemotherapy-induced premature ovarian failure

Chemotherapy-induced premature ovarian failure refers to ovarian failure occurring after chemotherapy treatment (Molina et al., 2005). The ovarian damage caused by chemotherapy is a common treatment complication among female cancer patients and affects their quality of life (Chen and Manson, 2006). Discovering ovarian protective agents with minimal side effects for clinical use could dramatically enhance the quality of life for female cancer patients undergoing chemotherapy, offering substantial social and academic advantages. Studies have indicated that GM influences the host’s responsiveness to chemotherapy drugs, enhancing efficacy and neutralizing drug toxicity. This suggests that GM will be a key aspect of individualized treatment for cancer patients (Zhang et al., 2016). Gori et al. (2019) found that antibiotics against Gram-positive bacteria can reduce the therapeutic effect of platinum-based chemotherapy drugs; Perales-Puchalt et al. (2018) discovered that fecal microbiota transplantation from healthy mice could improve Cis-platinum-induced intestinal mucosa damage. Research indicates that cyclophosphamide-induced POF in mice leads to notable alterations in gut microbiota, characterized by reduced *Akkermansia* abundance and increased *Lactobacillus* levels. Fisetin mitigates CTX-induced ovarian damage by modulating the GM to decrease CD4 + T lymphocytes and IL-12 levels (Lin et al., 2020). Targeting the GM could be a “breakthrough” in studying the pathogenesis of CIPOF and developing new preventive and therapeutic drugs.

## 6 Conclusion

Premature ovarian failure is closely related to GM dysfunction, which not only affects disease progression but also exacerbates complications such as mental and psychological problems, decreased bone density, and autoimmune diseases (Table 1). Although the importance of GM and its metabolites in the pathogenesis of POF is increasingly recognized, current research still faces many challenges. These include the lack of long-term human studies, insufficient understanding of GM functional changes, and the wide variation in microbial profiles due to the diverse etiologies of POF. Future research should focus on longitudinal microbiome analysis in POF patients to uncover the deep connections and potential causal mechanisms between GM and POF. Integrating microbiome analysis into POF’s diagnostic and therapeutic strategies is essential for more precise and personalized treatments. Given that current studies have only demonstrated an association between GM and POF, further validation and exploration of their causal relationship are needed through large-scale samples, multicenter clinical trials, and animal experiments. Future research directions include using liquid chromatography-mass spectrometry (LC-MS) to analyze metabolites and their pathways in the serum and fecal samples of POF patients and healthy controls; integrating multi-omics data from microbiomics, genomics, and transcriptomics to construct comprehensive models that reveal the complex relationship between GM dysbiosis and POF, and identify new biomarkers and therapeutic targets; and conducting cell culture and animal model experiments to verify the impact of GM metabolites on ovarian and granulosa cell functions, thereby understanding the interaction mechanisms between GM and POF at the molecular level. These research directions and methods will provide new ideas and strategies for diagnosing and treating POF.

**TABLE 1:** Changes in the gut microbiota associated with ovarian dysfunction.

Disease	Sample source	Main findings	References
Ovariectomy	Mice	Gnotobiotic mice that received the gut microbiome from ovariectomized mice fed the low-fat diet had greater weight gain and hepatic gene expression related to metabolic dysfunction and inflammation than those that received intact sham control-associated microbiome.	Cross et al., 2024
Ovariectomy	Mice	Microbiota-based intervention to delay or reserve ovarian aging is an appealing approach and may offer new therapeutic strategies for intestinal microbiota regulation to improve female fertility.	Huang et al., 2024
POI	Human	*E. hallii* and *E. ventriosum* have protective effects against POI, whereas *Intestinibacter* and *Terrisporobacter* have detrimental effects on POI	Wang et al., 2023
POI	Human	Phylum Bacteroidetes, genera *Butyricimonas*, *Dorea*, *Lachnobacterium* and *Sutterella* enriched significantly in women with POI	Wu et al., 2021
POF	Mice	The proportions of *Helicobacter*, *Odoribacter*, and *Alistipes* were lower in the dominant flora of the POF group, while *Clostridium XIVa*, *Barnesiella*, *Bacteroides*, and *Mucispirillum* were higher	Cao et al., 2020
POF	Mice	During CTX induced POF, the abundance of *Akkermansia* decreased while the abundance of *Lactobacillus* increased	Lin et al., 2020
POI	Human	Higher *Butyricimonas*, *Dorea*, *Lachnobacterium*, and *Sutterella* and lower *Bulleidia* and *Faecalibacterium* abundances at the genus level were observed in women with POI	Jiang et al., 2021
